# Positive non-sentinel axillary lymph nodes in breast cancer with 1-2 sentinel lymph node metastases

**DOI:** 10.1097/MD.0000000000013015

**Published:** 2018-11-02

**Authors:** Jianwei Zheng, Shuyan Cai, Huimin Song, Yunlei Wang, Xiaofeng Han, Haoliang Wu, Zhigang Gao, Fanrong Qiu

**Affiliations:** Department of General Surgery, Beijing Chaoyang Hospital Affiliated with Capital Medical University, Beijing, China.

**Keywords:** breast cancer, invasive tumor size, lymphovascular invasion, non-sentinel lymph node, risk factors, sentinel lymph node biopsy

## Abstract

Recent clinical trials have shown that sentinel lymph node biopsy (SLNB) alone without axillary lymph node dissection (ALND) can offer excellent regional control if there is sentinel lymph nodes (SLN) metastases to 1-2 nodes. This study aimed to explore the predictive factors for non-sentinel lymph node (NSLN) metastasis in breast cancer patients with 1-2 positive SLNs.

Patients with breast cancer and 1-2 positive SLN admitted between March 2009 and March 2017 and who underwent ALND after SLN biopsy (SLNB) at Beijing Chaoyang Hospital were analyzed retrospectively. Factors influencing the status of NSLN were studied by univariate and multivariate analysis.

Of 1125 patients, 147 patients had SLN metastasis (13.1%) and 119 patients (81.0%) had 1-2 positive SLNs. Among them, 42 patients (35.3%) had NSLN metastasis. The invasive tumor size (*P* <.001), histological grade (*P* =.011), lymphovascular invasion (LVI) (*P* =.006), and over-expression of HER2 (*P* =.025) significantly correlated with non-SLN metastasis by univariate analysis. LVI (LVI) (*P* =.007; OR: 4.130; 95% confidence interval [CI]: 1.465–11.641), invasive tumor size (*P* <.001; OR: 7.176; 95% CI: 2.710–19.002), and HER2 over-expression (*P* =.006; OR: 5.489; 95% CI: 1.635–18.428) were independently associated with NSLN metastasis by the Logistic regression model. The ROC analysis identified a cut-off point of 26 mm of tumor size (area under the receiver operating characteristic [ROC] curve [AUC] 0.712, CI: 0.614–0.811) was useful for dividing patients with positive SLN (1-2 nodes) into non-SLN-positive and non-SLN-negative groups.

For 1-2 positive SLNs of breast cancer, LVI, large invasive tumor size, and HER2 over-expression are independent factors affecting NSLN metastases.

## 1 Introduction

Axillary lymph node dissection (ALND) is the standard staging and therapeutic procedure for preoperatively diagnosed node-positive breast cancer.^[1]^ Current practice guidelines recommend a complete ALND for breast cancer patients whose sentinel lymph node (SLN) contains metastatic tumor, but complete ALND is associated with substantial morbidity affecting up to 39% of patients, with a nearly 3-fold increased risk of lymphedema or regional sensory loss.^[2]^

In the 1990s, the introduction of sentinel lymph node biopsy (SLNB) has resulted in changes in the management of the axilla.^[3]^ SLNB has become the standard of care as staging procedure in clinically node-negative breast cancer patients because 40% to 60% of SLN-positive patients do not have non-sentinel lymph node (NSLN) involvement.^[4]^

Although ALND provides additional prognostic information such as the number of involved lymph nodes and may decrease the rate of regional recurrence, its impact on survival seems negligible coupled with an increased morbidity as compared to SLNB.^[5]^

Recent clinical trials have suggested that there is no difference in the outcomes of patients with positive SLN whether they are treated with ALND or given no further axillary surgery.^[5,6]^ These studies raised doubts concerning the role of SLNB. A recent trial compared SLNB with the assessment of whether an axillary ultrasound is negative in patients with early breast cancer.^[7]^ SLN metastasis is observed in approximately 30% of SLNBs.^[8]^ Various clinicopathological parameters have been identified as independent predictors of axillary lymph node metastasis in early breast Cancer:^[9]^ clinical palpability,^[10]^ invasive carcinoma size,^[11]^ lymphovascular invasion (LVI),^[10]^ histological grade,^[11]^ hormone receptor (HR) status,^[12]^ age,^[13]^ multifocality, metastasis detection method, number of positive and negative SLN, and molecular subtype classification.^[14]^ According to the American College of Surgeons Oncology Group (ACOSOG) Z0011 trial, among patients with ≥2 positive SLNs, breast cancer treated with breast conservation and systemic therapy, SLNB did not result in inferior survival compared with ALND (5-year overall survival was 92.5% vs 91.8%; 5-year disease-free survival was 83.9% vs 82.2%).^[5]^ The International Breast Cancer Study Group Trial 23–01 (IBCSG 23–01)^[7]^ indicated that ALND should be avoided if metastases are detected in only 1 or 2 SLNs. On the other hand, SLN-positive patients will receive systemic therapy regardless of the presence of any additional nodal metastasis; therefore, the therapeutic impact of the number of the positive lymph nodes is minimal.^[15]^

Nevertheless, in 40% to 60% of patients, the NSLNs contain no further metastases and ALND provides no benefit, meaning that these patients underwent an unnecessary ALND.^[5]^ In addition, the incidence of regional recurrence is much lower than expected when axillary surgery was omitted.^[16]^ Therefore, the ability of a diagnostic test to predict the chance of NSLN involvement is critical to avoid unnecessary ALND. Giuliano et al^[17]^ reported that SLNB alone does not result in decreasing survival in SLN-positive patients. A recent review concluded that there is a potential role for avoiding ALND in selected SLN-positive patients.^[18]^ In studies using different techniques, the possibility of a NSLN metastasis in SLN-negative patients (false-negative) is reported to be 0% to 11%; this rate increases to 12% to 14% if isolated tumor cells (ITCs) are detected in the SLN, and to 20% to 35% in the presence of micrometastasis.^[19]^ Hence, factors that could predict the NSLN status in the presence of SLN involvement were examined and numerous pathological factors were found to be associated with higher NSLN involvement in the presence of a positive SLNB.

Management strategies that avoid invasive ALND are needed for node-negative patients. If we can predict the state of the axillary lymph nodes before SLNB, individuals who are node-negative or limited node-positive could avoid the unnecessary SLNB or ALND. Unfortunately, the preoperative clinical and imaging examinations of the axillary are rather poor for predicting axillary lymph node metastasis.

Therefore, the goal of this study was to characterize the various clinicopathological features of patients with invasive breast cancer in order to identify the risk factors that might help in predicting positive NSLN, particularly in cases of SLN metastasis in 1 to 2 nodes, hereby exploring the feasibility of avoiding ALND.

## 2 Materials and Methods

### 2.1 Patients

Patients with invasive breast cancer who underwent SLNB at the Beijing Chaoyang Hospital affiliated with Capital Medical University between March 2009 and March 2017 were eligible for inclusion in this retrospective chart review. Invasive breast cancer was defined as penetration of the basement membrane by malignant cells invading the stroma.^[20]^ ALND was performed in patients for no clinically positive axillary lymph nodes with macro- and micrometastasis in the SLNs. Of the patients with positive SLN, those with 1 or 2 positive SLNs were evaluated further. Only those patients with complete data for clinicopathological factors including age, primary tumor size (in cm), histological type (ductal/lobular), histological grade (I–III), LVI, venous involvement, tumor location, hormonal receptor status, human epidermal growth factor receptor 2 (HER2) status, molecular subtype, Ki-67 index, and menstruation status were enrolled in the study. There were 1125 patients who satisfied the following criteria:

1) diagnosis of breast cancer by core needle aspiration or excisional biopsy;
2) no significant palpation of axillary lymph nodes;
3) no preoperative anti-cancer therapy;
4) preoperative SLNB performed; and
5) ALND.

The exclusion criteria were:

1) without SLN metastasis;
2) >2 positive SLNs; or
3) ≤2 total number of SLNs. The numbers of positive and negative nodes on ALND were recorded.

Ethical approval for this study was obtained from Chaoyang Hospital Research Ethics Committee. Individual informed consent was waived by the committee because of the retrospective nature of the study.

### 2.2 SLN biopsy

The technique of SLNB was adapted to early breast cancer.^[21]^ SLNs were identified after peri-areolar intradermal injection of carbon nanoparticles (Chongqing LUMMY Pharmaceutical Co., Chongqing, China) in the form of a standard carbon nanoparticle suspension (1 mL = 50 mg). Nanoparticle suspension (1 mL) was intradermally injected into the periareolar region or glandular tissue around the tumor in the 4 (clockwise) quadrants of the breast 10 minutes before surgery. The whole breast was massaged for about 5 minutes to facilitate the absorption of the carbon nanoparticles into the lymph vessels. In order to identify the stained lymph nodes, a transverse incision was made just below the hair-bearing region of the axilla. After raising the skin flaps, black stained lymphatic tracts were meticulously searched and traced towards the axilla. The lymph nodes stained black and to which a black lymphatic trace led were considered as SLNs. The SLNB was also performed by using blue dye 10 minutes before operation in some patients. A 1% solution of blue dye (1 mL) was injected in the same way as the carbon nanoparticles and the axilla was explored in the same manner. The lymph nodes stained blue were considered as SLNs. All SLNs were harvested and sent for pathological examination. Intraoperative frozen section was performed on all SLNs. The SLN was cut longitudinally into 2 halves. Half of the node was frozen for immediate examination, and up to 2 sections were stained with hematoxylin and eosin (H&E). The other half was fixed in formalin and embedded in paraffin, and up to 2 sections were stained with H&E.

The size of the metastasis was categorized according to the American Joint Committee on Cancer (AJCC) Staging Manual (7th edition): ITCs were defined as a metastasis ≤0.2 mm (pN0i+); micrometastases (MI) were defined as >0.2 mm but ≤2 mm (pN1mi), and macrometastases were defined as >2 mm (pN1). A sentinel node was defined as positive in the presence of a macrometastasis, micrometastasis, or ITC.

All procedures of SLNB were completed within 30 to 45 minutes. Due to inherent cultural barriers and cancer fatalism in Chinese women, all patients chose complete ALND upon diagnosis of breast cancer. The results of ALND were used for validating the results of SLNB. For all additional nodes identified by ALND, routine H&E analysis was conducted on a single section of each node.

### 2.3 Histopathological evaluation

The fresh tissue containing the SLN (1 or more fragments) was submitted for intraoperative pathological examination by cytological imprinting and frozen section. Immunohistochemistry (IHC) for estrogen receptor (ER) and progesterone receptor (PR) status was performed and tumors were deemed positive for each receptor if at least 10% of the invasive tumor cells in a section exhibited nuclear staining. HER2 expression was examined by IHC, which was determined according to the category (−, 180 +, ++, and +++). HER2 over-expression was defined as 3+ on IHC, or 2+ on IHC and positive with fluorescence in situ hybridization (FISH). FISH was used in cases when it was difficult to determine the HER2 status by IHC. Ki-67 was examined by IHC, and the results were expressed as the percentage of stained tumor cells.^[22]^

Hematoxylin-eosin staining was used to assess LVI and histological grading, which was defined according to the Scarff-Bloom-Richardson system.^[23]^ SLNs were re-examined postoperatively using fixed sections. All nodal structures isolated were serially cross-sectioned at 2-mm intervals perpendicular to the longitudinal axis. In cases in which the size of the SLN was <5 mm, bisection along the longest axis was considered acceptable.

Breast cancer was staged according to the TNM 7th edition classification, as proposed by the AJCC. All IHC evaluations were performed by 4 well-trained pathologists. In cases in which assessment of grade differed, the disagreements were solved by consensus after joint review using a conference microscope.

Patients were assigned into 4 subgroups, as proposed by the St Gallen International Expert Consensus^[24]^ based on the results of their ER, PR, and HER2 statuses, and Ki-67 index:^[25]^

1) luminal A, that is, ER or PR positive, HER2 negative, and Ki-67 index <14%;^[23]^
2) luminal B, that is, ER or PR positive, HER2 positive, or Ki-67 index ≥14%;
3) HER2-positive, that is, ER and PR negative and HER2 positive; and
4) triple-negative, that is, negative for ER, PR, and HER2. To obtain the overall tumor histological grade, the scores for each category were added together, giving a possible total of 3 to 9. Histological grade was then allocated on the following basis:^[26]^ 3 to 5 points: grade I, well-differentiated; 6 to 7 points: grade II, moderately differentiated; and 8 to 9 points: grade III, poorly differentiated. This method of assessing tumor differentiation (along with most other methods) is based essentially on subjective assessment of morphological features.

### 2.4 Statistical analysis

The associations between NSLN metastasis and clinicopathological factors were examined. Descriptive statistics were reported for all variables. Continuous variables were presented as median (range). Categorical variables were presented as frequencies and percentage. Statistical analyses were performed using the Mann–Whitney's *U* test and the Chi-square test. Univariate logistic regression was performed. Variables with *P* values <.05 in univariate analyses were included in the multivariate logistic regression (forward) analysis. The diagnostic accuracy of the invasive tumor size was assessed by receiver operating characteristic (ROC) analysis.

The area under the ROC curve (AUC) with 95% confidence interval (CI) was used to assess model discrimination. It is generally accepted that AUC values 0.7 to 0.8 represent fair discrimination, whereas AUC values >0.8 represent good discrimination. Statistical significance was set at *P* <.05. SPSS 20.0 (IBM, Armonk, NY) was used for statistical analyses.

## 3 Results

### 3.1 Positive SLNs and NSLNs

**Figure 1:**
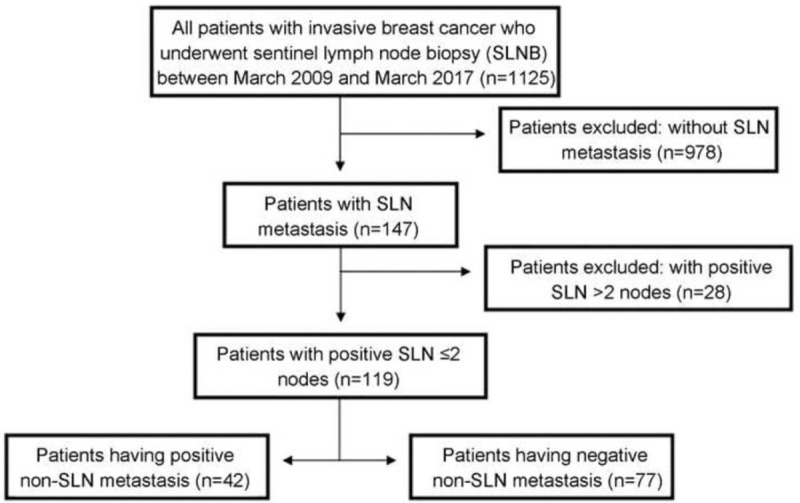
Study flowchart.

A total of 1125 patients were included. All patients were female. Among the 1125 patients, 147 had positive SLN (13.1%), while 978 had negative SLN (86.9%). The median of SLNs sampled for each patient was 2.9 (range, 1–10). Of the patients with positive SLN, 1 to 2 positive SLNs and a total of ≥3 SLNs were observed in 119 patients (81.0%); they met the inclusion criteria and received routine ALND. Among these 119 patients, NSLN metastasis was observed in 42 patients (35.3%) with an average of 16.5 NSLN for each patient. Seventy-two patients (60.5%) had 1 positive SLN, 26 patients (21.8%) had 2 positive SLNs, and 21 patients (17.7%) had ITCs in SLNs (Fig. 1).

### 3.2 Patient characteristics and clinicopathological factors

**Figure 2:**
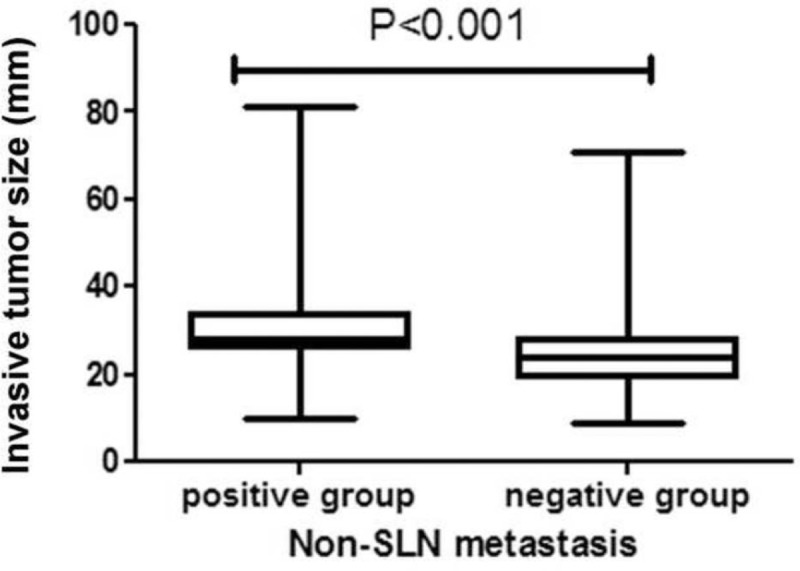
Association between invasive tumor size and NSLN metastasis (n = 119). Invasive tumor size was significantly larger in patients with positive NSLN compared with those who were negative (median size 28 vs 24 mm, respectively, *P* <.001). NSLN = non-sentinel lymph node.

**Figure 3:**
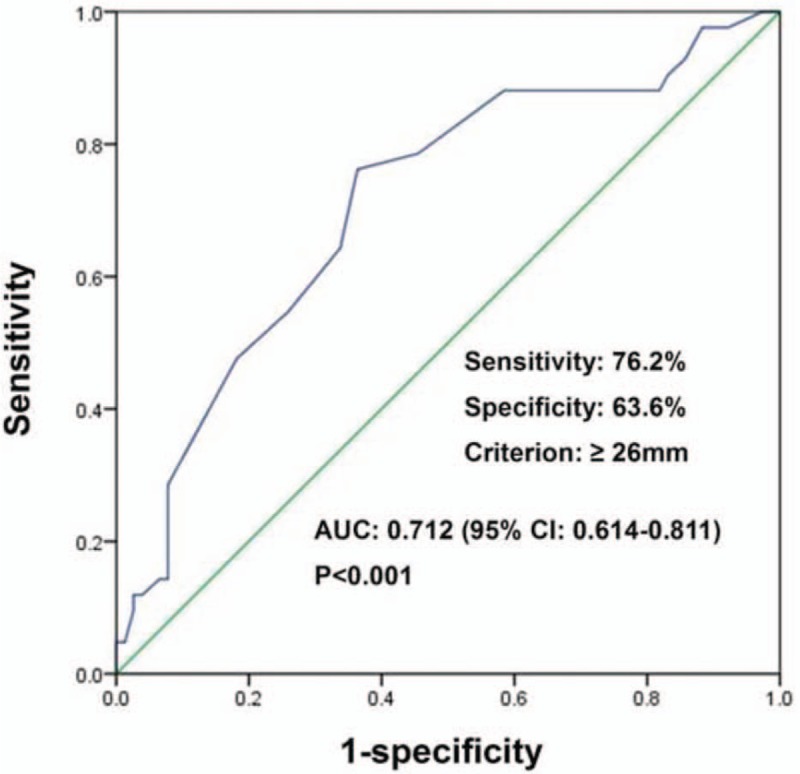
ROC analysis of invasive tumor size (n = 119). The ROC analysis identified a cut-off point of 26 mm (area under the curve 0.712; sensitivity 76.2%; specificity 63.6%; *P* <.001). ROC = receiver operating characteristic.

Median patient age was 52 years (range 28–75 years). There was no association between age and non-SLN metastasis (Table 1). Univariate analyses revealed that non-SLN metastasis was significantly association with histological grade (*P* =.011), LVI (*P* =.006), and HER2 expression (*P* =.004). Median invasive tumor size was significantly larger in patients with positive NSLN compared with patients with negative NSLN (28 vs 24 mm, *P* <.001) (Fig. 2). Therefore, we investigated the threshold value for invasive tumor size that differentiated patients with NSLN metastasis using ROC analysis. ROC analysis identified a cut-off point of 26 mm 246 (AUC 0.712, CI: 0.614–0.811; false-negative rate of 23.8%; 76.2% sensitivity; 63.6% specificity; positive predictive value of 53.3%; negative predictive value of 83.1%; 68.1% accuracy; *P* <.001; Fig. 3). We then used this cut-off point of 26 mm to assess the patients. As shown in Table 2, using a cut-off value of 26 mm resulted in a significant association between invasive tumor size and NSLN metastasis (P <.001), but there was no significant association between non-SLN metastasis and age, histological type, tumor location, menstruation, venous involvement, molecular subtype, ER and/or PR status, or Ki-67 labeling index.

**Table 1:**
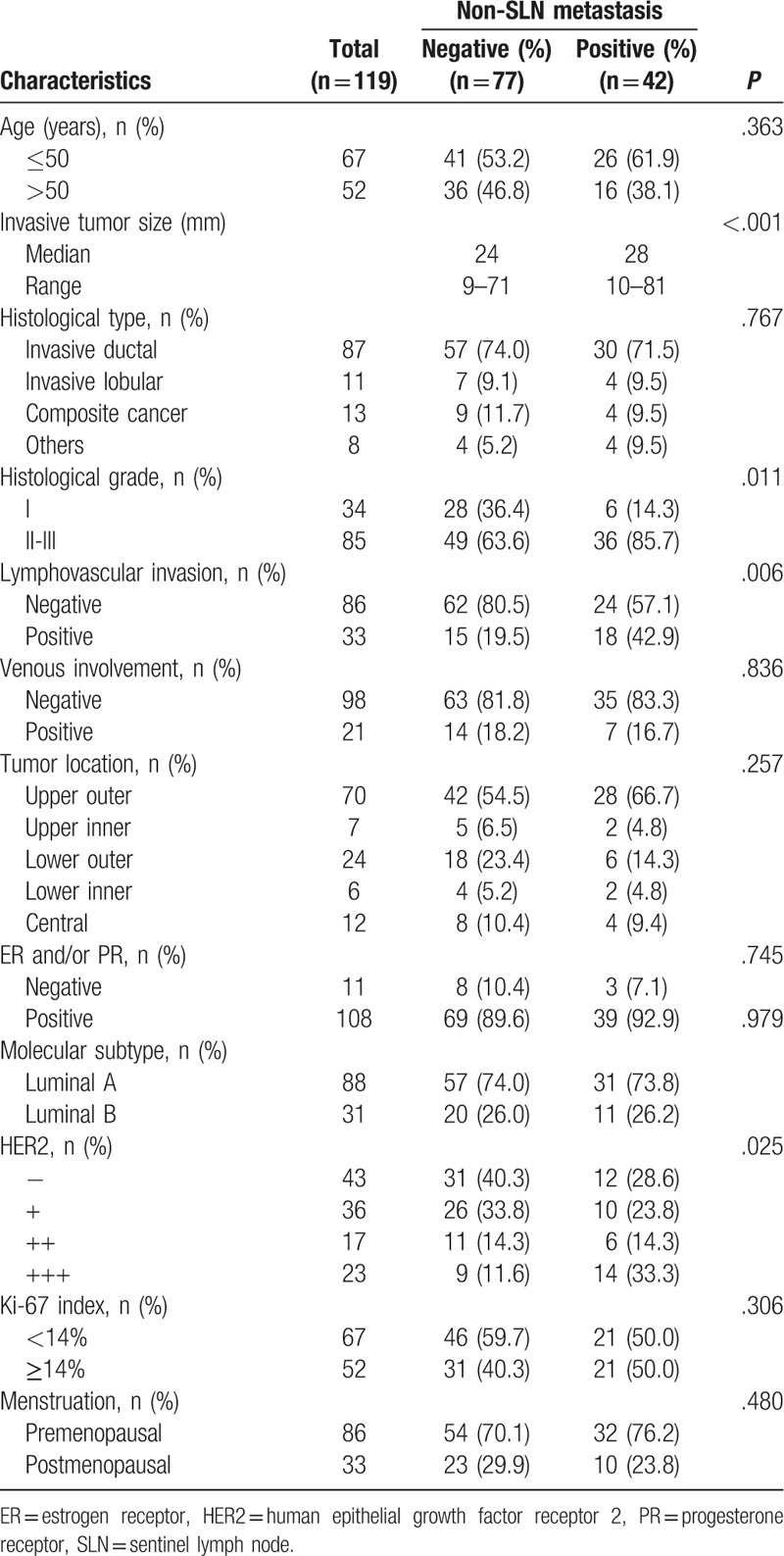
Association between non-sentinel lymph node metastasis and clinicopathological features.

**Table 2:**
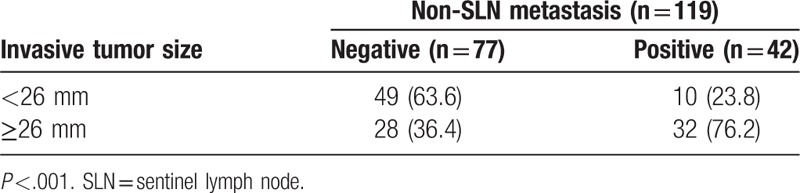
Non-sentinel lymph node metastasis and cut-off points of invasive tumor size.

### 3.3 Multivariate analysis of predictor for NSLN metastasis

Multivariate analysis showed that LVI (*P* =.007; OR: 4.130; 95% CI: 1.465–11.641), 257 invasive tumor size (*P* <.001; OR: 7.176; 95% CI: 2.710–19.002), and HER2 expression (*P* =.006; OR: 5.489; 95% CI: 1.635–18.428) were independently associated with NSLN metastasis (Table 3). Histological grade was not independently associated with NSLN metastasis when all 4 variables were considered together (*P* =.472).

**Table 3:**
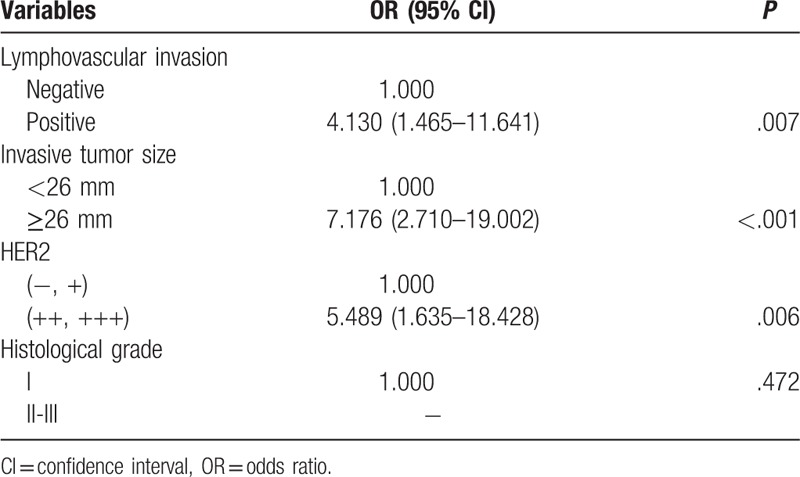
Multivariate analysis of clinicopathological factors (n = 119).

## 4 Discussion

SLNB alone without ALND can offer excellent regional control if there is ≤2 positive SLNs. This study aimed to explore the characteristics and predictive factors for NSLN metastasis in breast cancer patients with 1-2 positive SLNs and examine the factors associated with positive NSLN. The results suggest that for 1-2 positive SLNs, LVI, large invasive tumor size, and HER2 over-expression are independently associated with NSLN metastases.

SLNB for clinically node-negative breast cancer has become a standard procedure worldwide and it is important to determine pathologically whether the node is negative before surgery. Although ALND has been the standard axilla management approach for patients with positive SLN,^[9]^ postoperative pathology examination showed that some SLN-positive patients had no NSLN metastasis. Recent clinical trials suggest that ALND is unnecessary if a metastasis is detected in 1 or 2 SLNs.^[^5,17] Van la Parra et al^[27]^ found that in patients with clinically node-negative disease, the SLN was the only involved node in 40% to 60% of patients undergoing SLNB. The ACOSOG Z0011 study^[5]^ showed that patients with H&E-detected metastases in the SLN would have similar outcomes whether they were randomized to ALND or no ALND and no axillary irradiation. SLNB alone without ALND results in extremely low locoregional recurrence and excellent overall survival, comparable to that in patients undergoing ALND if they have 1-2 positive SLN. No significant benefit in locoregional control was seen with ALND despite the removal of additional positive lymph nodes. Removal of additional positive nodes with ALND did not result in fewer locoregional recurrences than did SLND alone at a median follow-up of 6.3 Years.^[5]^ In the present study, we investigated patients with 1-2 positive SLNs to elucidate the clinicopathological factors associated with NSLN. The results revealed that 42 (35.3%) patients had NSLN metastasis among the 119 patients with 1-2 positive SLNs, which is similar to a Japanese study.^[28]^ Therefore, breast surgeons must accept the risk of remaining NSLN metastasis if they do not perform ALND for patients with 1-2 SLN metastases. Thus, the prediction of NSLN metastasis in the case of 1-2 positive SLN metastasis is necessary when making decisions regarding an additional ALND.

Previous studies have reported that younger age, higher pT stage, and LVI were independent predictors of SLN metastasis, with hormonal receptors and histological grade being good predictors of positive SLN.^[10,28]^ With regard to the prediction of NSLN metastases, younger age, large tumor size or higher pT stage, LVI, extracapsular invasion, the ratio of positive SLNs to the total number of harvested SLNs, or total tumor load in the SLNs assessed by 1-step nucleic acid amplification have been reported as useful markers.^[29]^ Nevertheless, these studies were not performed in patients with 1–2 positive SLNs. Therefore, in the present study, we investigated the association between NSLN metastasis and clinicopathological factors, particularly in patients with 1-2 positive SLNs. The univariate analyses revealed that invasive tumor size, histological grade, LVI, and HER2 over-expression were significantly associated with NSLN metastasis in patients with 1-2 SLN metastasis. Invasive tumor size was significantly larger in patients who had positive NSLN compared with those who had no positive NSLN. The ROC analysis identified 26 mm as the best cut-off point to discriminate between positive and negative NSLN patients. The multivariate analysis showed that invasive tumor size, LVI, and HER2 over-expression were independently associated with NSLN metastasis. Therefore, these results suggest that in the presence of invasive tumor ≥26 mm, LVI, and over-expressed HER2, there is a higher probability of NSLN metastases.

Over the past years, several studies have been conducted to identify clinicopathological variables predictive of NSLN metastases in order to select patients who would benefit the most from ALND. These studies demonstrated that different pathological characteristics of the primary tumor and SLNs were associated with an increased possibility of positive NSLN. Nevertheless, there is no consensus on the predictive factor of NSLN metastasis.^[30]^ Our results were consistent with previous studies in that tumor size and LVI were associated with NSLN metastasis^[28]^ except HER2 over-expression. Kwon et al^[30]^ indicated that there is no significant relationship between HER2 expression and NSLN metastasis. In contrast, the results obtained in this study showed that over-expression of HER2 significantly increased the probability of NSLN metastasis. HER2 is well known for its ability to enhance cell proliferation, survival, motility, and adhesion.^[31]^ HER2 over-expression induces lymphangiogenesis, promotes metastasis, and results in poor prognosis of breast cancer by up-regulating vascular endothelial growth factor-C (VEGF-C) expression.^[32]^

A study by Schoppmann et al^[33]^ showed that over-expression of HER2 would increase the probability of tumor metastasis and that inhibiting Her-2/neu may reduce tumor progression by blocking VEGF-C-mediated tumor cell proliferation and lymphogenic metastasis.

According to the guidelines of the American Society of Clinical Oncology/College of American Pathologists for HER2 testing in breast cancer, HER2 – and + are HER2-negative. HER2 +++ is positive. When HER2 IHC is equivocal (++), the HER2 status has to be confirmed by FISH.^[34]^ In the present study, we analyzed the difference of NSLN metastasis between HER2 – and + versus HER2 ++ and +++. In patients with HER2 – and +, the probability of additional NSLN metastases was 27.8% (22/79) compared with 50% (20/40) for patients with HER2 ++ 342 and +++.

Recently, many mathematical models have been developed to evaluate the predictive factor of NSLN metastasis in SLN-positive patients, including the Memorial Sloan Kettering Cancer Center (MSKCC) chart, Tenon score, Stanford model, Cambridge model, and Mayo algorithm. Various indicators in these studies may lead to poor accuracy of specific patient populations. Terrier et al^[35]^ found that the MSKCC nomogram and Tenon score model are better than the other models. Another multi-center study^[36]^ found that the AUC of the Tenon score was only 0.582, while the MSKCC nomogram, Cambridge model, and Stanford model had AUCs of 0.705, 0.711, and 0.730, respectively. The Shanghai Cancer Center Non-SLN nomogram showed an AUC of 0.78.^[37]^ Therefore, large-scale multi-center clinical studies are still needed to improve the above models.

The main limitations of the present study include its retrospective nature and the small number of patients who had positive SLN. We believe, however, that these limitations did not greatly affect the results of the study as the differences between NSLN positive and negative patients were too marked to have resulted from bias. Our results thus provide useful information on the risk factors for remnant NSLN metastasis under the condition of 1-2 positive SLNs. Prospective studies with larger number of patients are necessary to achieve an explicit standard for SLN-positive patients to avoid ALND, or evaluate appropriate treatment for patients with positive SLNs (1-2). The validation of these predictors in prospective studies may enable approximately half of early breast cancer patients with positive SLN to be staged with SLNB alone while avoiding the morbidity of unnecessary ALND.

## 5 Conclusion

NSLN metastasis was found in more than 30% of patients. Even in the presence of 1-2 positive SLNs, large tumor size (>26 mm), the presence of LVI, and HER2 over-expression were significantly associated with non-SLN metastasis.

## Acknowledgments

The authors wish to thank the patients and their families for participating in the study.

## Author contributions

**Conceptualization:** Fanrong Qiu.

**Data curation:** Huimin Song.

**Investigation:** Yunlei Wang.

**Methodology:** Zhigang Gao.

**Project administration:** Xiaofeng Han.

**Visualization:** Haoliang Wu.

**Writing – original draft:** Jianwei Zheng.

**Writing – review & editing:** Shuyan Cai.
